# *Listeria monocytogenes* Induces a Virulence-Dependent microRNA Signature That Regulates the Immune Response in *Galleria mellonella*

**DOI:** 10.3389/fmicb.2017.02463

**Published:** 2017-12-12

**Authors:** Gopala K. Mannala, Benjamin Izar, Oliver Rupp, Tilman Schultze, Alexander Goesmann, Trinad Chakraborty, Torsten Hain

**Affiliations:** ^1^Institute of Medical Microbiology, Justus-Liebig University, Giessen, Germany; ^2^Department of Medical Oncology, Dana-Farber Cancer Institute and Harvard Medical School, Boston, MA, United States; ^3^Broad Institute of MIT and Harvard University, Cambridge, MA, United States; ^4^Department of Bioinformatics and Systems Biology, Justus-Liebig University, Giessen, Germany

**Keywords:** *Galleria mellonella*, *Listeria monocytogenes*, *Listeria innocua*, infection, microRNA, miRNA, immune response

## Abstract

microRNAs (miRNAs) coordinate several physiological and pathological processes by regulating the fate of mRNAs. Studies conducted *in vitro* indicate a role of microRNAs in the control of host-microbe interactions. However, there is limited understanding of miRNA functions in *in vivo* models of bacterial infections. In this study, we systematically explored changes in miRNA expression levels of *Galleria mellonella* larvae (greater-wax moth), a model system that recapitulates the vertebrate innate immunity, following infection with *L. monocytogenes*. Using an insect-specific miRNA microarray with more than 2000 probes, we found differential expression of 90 miRNAs (39 upregulated and 51 downregulated) in response to infection with *L. monocytogenes*. We validated the expression of a subset of miRNAs which have mammalian homologs of known or predicted function. In contrast, non-pathogenic *L. innocua* failed to induce these miRNAs, indicating a virulence-dependent miRNA deregulation. To predict miRNA targets using established algorithms, we generated a publically available *G. mellonella* transcriptome database. We identified miRNA targets involved in innate immunity, signal transduction and autophagy, including spätzle, MAP kinase, and optineurin, respectively, which exhibited a virulence-specific differential expression. Finally, *in silico* estimation of minimum free energy of miRNA-mRNA duplexes of validated microRNAs and target transcripts revealed a regulatory network of the host immune response to *L. monocytogenes*. In conclusion, this study provides evidence for a role of miRNAs in the regulation of the innate immune response following bacterial infection in a simple, rapid and scalable *in vivo* model that may predict host-microbe interactions in higher vertebrates.

## Introduction

microRNAs (miRNAs) are small endogenous non-coding RNA molecules with a mature size of 22 nucleotide that regulate gene expression on a post-transcriptional level by binding the 3′ UTR of their target mRNA and thereby leading to its degradation or translation inhibition (Bartel, 2004; He and Hannon, 2004; Krol et al., 2010). miRNAs are involved in the control of several physiological and pathological processes, such as immunity, apoptosis, carcinogenesis, and cardiovascular diseases (Ambros, 2004). Recently, a number of reports described a role of miRNAs in host-pathogen interactions in models of viral and bacterial infections of a range of hosts. Various conceptual mechanisms of bacteria-mediated miRNA expression alteration in host cells were established in the last years. These include physical interaction of flagellin with a cell surface receptor (Navarro et al., 2008), cell invasion (Schulte et al., 2011), secretion of virulence factors and others (Eulalio et al., 2012; Staedel and Darfeuille, 2013). Recent studies demonstrated that infection with *L. monocytogenes* alters the miRNA profile and the expression of targeted mRNAs that regulate the host immune response (Ma et al., 2011; Schnitger et al., 2011; Izar et al., 2012). In addition, probiotic strains such as *Lactobacillus casei, Lactobacillus paracasei* and gut microbiota interfere with miRNA response of mice that are with orally acquired listeriosis, whereas intestinal microbiota provides insight about the *in vivo* microRNA response due to listerial infection (Archambaud et al., 2012, 2013). The causative agent of listeriosis, *L. monocytogenes* is a gram-positive food-borne pathogen that causes a highly lethal systemic infection in animals and immune deficient humans (Hamon et al., 2006). The pathophysiology depends upon a variety of virulence factors that, in concert, result in a systemic infection of vulnerable host organisms. After consumption of contaminated food, the bacterium crosses the epithelial barrier of the gut using internalins and subsequently escapes from phagocytic vacuole by the pore forming listeriolysin and phospholipases. The greater wax moth *Galleria mellonella* is a powerful model system to study the pathogenesis and virulence of several microbial pathogens, including *L. monocytogenes* and for high-throughput screening of its mutants (Mukherjee et al., 2010). In insects, the endosymbiont bacterium *Wolbachia* has been shown to induce the expression of aae-miR-2940 in mosquitoes, which targets the metalloproteinase and cytosine methyl- transferase genes and thereby plays a major role in bacterial maintenance (Zhang et al., 2013). As *G. mellonella* has been a prominent infection model organism to investigate various microbial pathogens, we took up a comprehensive study to reveal the miRNA profile and its role in immune regulation during *L. monocytogenes* infection in comparison to non-pathogenic *L. innocua* infection. Recently, Mukherjee et al. investigated the role of miRNAs in the different developmental stages of *G. mellonella* as well as in entomopathogenic fungal and bacterial infections, and demonstrated that miRNAs can act as mediators for trans-generational immune priming (Mukherjee and Vilcinskas, 2014), expanding the value of this *in vivo* model system.

In this study, we systematically elucidated the *in vivo* miRNA profile of *G. mellonella* larvae following infection with *L. monocytogenes* using a genome-wide insect specific miRNA microarray. Significant deregulation of a set of miRNA occurred exclusively in response to pathogenic *Listeriae* while non-pathogenic strains had little to no effect. To enable *in silico* target prediction, we generated a publically available *G. mellonella* transcriptome database. Virulence-dependent miRNAs were associated with differential expression of predicted target genes that are involved in the innate immune response and autophagy. Analysis of predicted minimum energy of miRNA-mRNA duplexes converged into a regulatory network that supports a role of miRNAs in host-microbe interactions. This study highlights the feasibility and scalability of *G. mellonella* as an *in vivo* model system to elucidate the role of miRNAs in bacterial infections.

## Materials and methods

### Insect and bacterial growth conditions

Conventional *G. mellonella* larvae were reared on artificial diet (22% maize meal, 22% wheat germ, 11% dry yeast, 17.5% bees wax, 11% honey, and 11% glycerin) at 30°C incubators before infection. Larvae in the last instar stage weighing ~150–200 mg were used for all experiments. We used 20 larvae for each experiment.

*Listeria monocytogenes* strain EGD-e (serotype 1/2a) and *L. innocua* CLIP 11262 (Glaser et al., 2001) were grown aerobically in BHI broth at 37°C at constant shaking at 180 rpm. For infection of the larvae, the overnight bacterial culture was diluted 1:50, grown to mid-exponential phase (OD_600nm_ = 1.0) and washed with 0.9% NaCl twice. Each larva was injected with 1 × 10^6^ CFU bacteria and incubated at 37°C for 7 days.

To determine the bacterial count in the infected larvae, the larvae were collected at 1st day and 5th day post infection. These larvae were ground in liquid nitrogen individually, dissolved in BHI broth with 1% Triton-X and incubated at room temperature for 5 min. Homogenate solution was serially diluted and plated out on *Listeria* specific PALCAM agar plates and incubated at 37°C for 2 days.

### Biosafety

All experiments were performed in biosafety level 2 (BSL2) laboratories approved and authorized for experimental work with *L. monocytogenes* by the local authorities (Regierungspraesidium Giessen:Az.IV44-53r30.03.UGI114.11.01/Az.IV44-53r30.03.UGI114.40.01/Az.IV44-53r30.03.UGI114.40.02).

### RNA isolation

On 5th day post infection, the larvae were ground well in liquid nitrogen, dissolved in Trizol solution, centrifuged at 8,000 × g for 15 min at room temperature and the supernatant was collected followed by addition of 100 μl of 1-bromo-3-chloropropane (BCP reagent, Molecular Research Centre, Inc). The sample was incubated at room temperature for 5 min, followed by incubation for 10 min on ice and centrifuged at 18,000 × g for 15 min at 4°C. The upper layer was transferred into fresh tube, pelleted by adding isopropanol and washed with 75% ethanol. Remaining DNA was digested with Turbo DNase (Ambion) and RNA was eluted by RNase free water. The RNA quantity was measured with Nano Drop analyzer (NanoDrop Technology, Rockland, MA, USA) and the quality was measured by Bioanalyzer 2100 (Agilent, Boeblingen, Germany).

### miRNA microarray

To construct the insect specific miRNA microarray, we collected miRNA sequence data from the miRNA registry Database (limited to miRNAs from Arthropods) (release 18; http://www.mirbase.org/). The miRNA microarray was constructed as standard protocols by the external provider (LC Sciences, Houston USA). For each miRNA microarray, we used 2 μg of total RNA from five surviving larvae out of 20, which were pooled for RNA isolation. Samples were 3′-extended with a poly (A) tail using poly adenylate polymerase. An oligonucleotide tag was then ligated to the poly (A) tail for later fluorescent dye staining; two different tags were used for the two RNA samples in dual-sample experiments. Hybridization was performed overnight on a Paraflo microfluidic chip using a micro-circulation pump (Atactic Technologies; Gao et al., 2004; Zhu et al., 2007). On the microfluidic chip, each detection probe consisted of a chemically modified nucleotide-coding segment complementary to the target miRNA (from miRBase, http://miRNA.sanger.ac.uk/sequences/) and a spacer segment of polyethylene glycol to extend the coding segment away from the substrate. The detection probes were made by *in situ* synthesis using PGR (photo-generated reagent) chemistry. The hybridization melting temperatures were balanced by chemical modifications of the detection probes. Hybridization used 100 μl 6xSSPE buffer (0.90 M NaCl, 60 mM Na_2_HPO_4_, 6 mM EDTA, pH 6.8) containing 25% formamide at 34°C. After RNA hybridization, tag-conjugating Cy3 and Cy5 dyes were circulated through the microfluidic chip for dye staining. Fluorescence images were collected using a laser scanner (GenePix 4000B, Molecular Device) and digitized using Array-Pro image analysis software (Media Cybernetics). Data was analyzed by first subtracting the background and then normalizing the signals using a LOWESS filter (Locally-weighted Regression; Bolstad et al., 2003). For two color experiments, the ratio of the two sets of detected signals (log2 transformed, balanced) and *p*-values of the *t*-test were calculated; detected signals with *p* < 0.01 were considered significantly differentially expressed. The total analyses of three independent experiments with log fold expression and statistical significance between control and infected larvae for each miRNA is available in Table S1.

### Data availability

Microarray data have been deposited to ArrayExpress (http://www.ebi.ac.uk/arrayexpress), accession number E-MTAB-4024.

### Reverse transcription and quantitative real-time PCR (qRT-PCR)

First strand cDNA synthesis was done for mRNA by using Super Script II reverse transcriptase (Invitrogen) and for miRNA miScript reverse transcription kit (Qiagen) was used. For both, 1 μg of total RNA was used as template. Quantitative real time PCR analysis was performed using the Step OnePlus Real-Time PCR System (Life Technologies). All the primers for real-time PCR were purchased from Qiagen, 18S rRNA was used as endogenous control for mRNA real-time PCR and endogenous controls for miRNA real-time PCR were selected based on expression stability in both infected and non-infected larvae (dme-miR-307a-3p used as endogenous control). For mRNA quantitative real-time PCR, 100 ng of cDNA and for quantification of miRNA 5 ng per reaction was used, respectively. The list of primers used for mRNA quantification and sequences used for miRNA quantification are listed in Table S2. Expression levels of miRNA and their target genes were determined by normalizing its quantity to the respective expression of internal controls in *G. mellonella*. The relative expression of these target genes were measured by using mathematical model for relative quantification of real-time PCR as described previously (Pfaffl, 2001).

### *Galleria mellonella* transcriptome database generation and target prediction

Publically available Illumina and 454 RNA-seq reads and ESTs from *G. mellonella* were retrieved from NCBI (SRR1021612, SRR1272440, ERR031115, ERR031116, ERR031117, ERR031118, ERR031119, ERR031120, ERR031121, and ERR031122). Additionally 18,690 pre-assembled contigs from an additional study (Vogel et al., 2011) were included. The read quality was checked using FastQC (Andrews, 2010) and trimmed accordingly (parameter used for Illumina reads: HEADCROP:15 ILLUMINACLIP:TruSeq3-PE-2.fa:2:30:10 MAXINFO:30:0.5 MINLEN:50, parameter used for 454 reads: HEADCROP:40 SLIDINGWINDOW:10:21 MINLEN:50 CROP:200 TOPHRED33) using Trimmomatic (Bolger et al., 2014). All reads were pooled and digitally normalized using the k-mer coverage approach implemented in Trinity (Haas et al., 2013). Multiple *de novo* assemblies were performed. The reads were assembled using the Trinity (Grabherr et al., 2011) assembler. The Velvet/Oases (Schulz et al., 2012) assembler was applied to assemble reads including the ESTs and pre-assembled contigs using the—*conserveLong* option to preserve the EST and pre-assembled contigs. To take into account the heterogeneity of the data, multiple Velvet/Oases assemblies were computed with varying k-mer parameters ranging from 19 to 75. The sequences from all *de novo* assemblies, the ESTs and pre-assembled contigs were screened for potential coding regions with Trans Decoder (transdecoder.github.io). The predicted amino acid sequences were clustered using cd-hit (Li and Godzik, 2006) with 98% global identity. For each cluster, the sequence with the longest 3′ UTR and a CDS length of at least 75% of the longest CDS in the cluster were selected as final transcripts. The transcripts were uploaded into SAMS (Rupp et al., 2014) and an automatic functional annotation was performed.

For miRNA target prediction only the 3′ UTR parts of the transcripts were used from the above prepared database. Target sites were predicted using miRanda (Enright et al., 2003) with *-strict* option to get only exact matching seed sequences. Gene ontology analysis was performed with GOstats (Falcon and Gentleman, 2007) applying default parameters. Using cytoscape, we created miRNA-mRNA network including target genes with known function. In addition, the minimum free energy level of miRNA-mRNA duplex structure was determined by RNAhybrid tool provided by Bielefeld Bioinformatics server (Rehmsmeier et al., 2004).

### Statistical analysis

Statistical analysis of experiments was performed with SigmaPlot 11 (Systat Software, San Jose, CA, USA). Student's *t*-test was used determine the significance of microRNA/mRNA expression levels. Survival data was analyzed using log rank test.

## Results

### Comprehensive miRNA expression profiling *G. mellonella* during infection with *L. monocytogenes*

**Figure 1:**
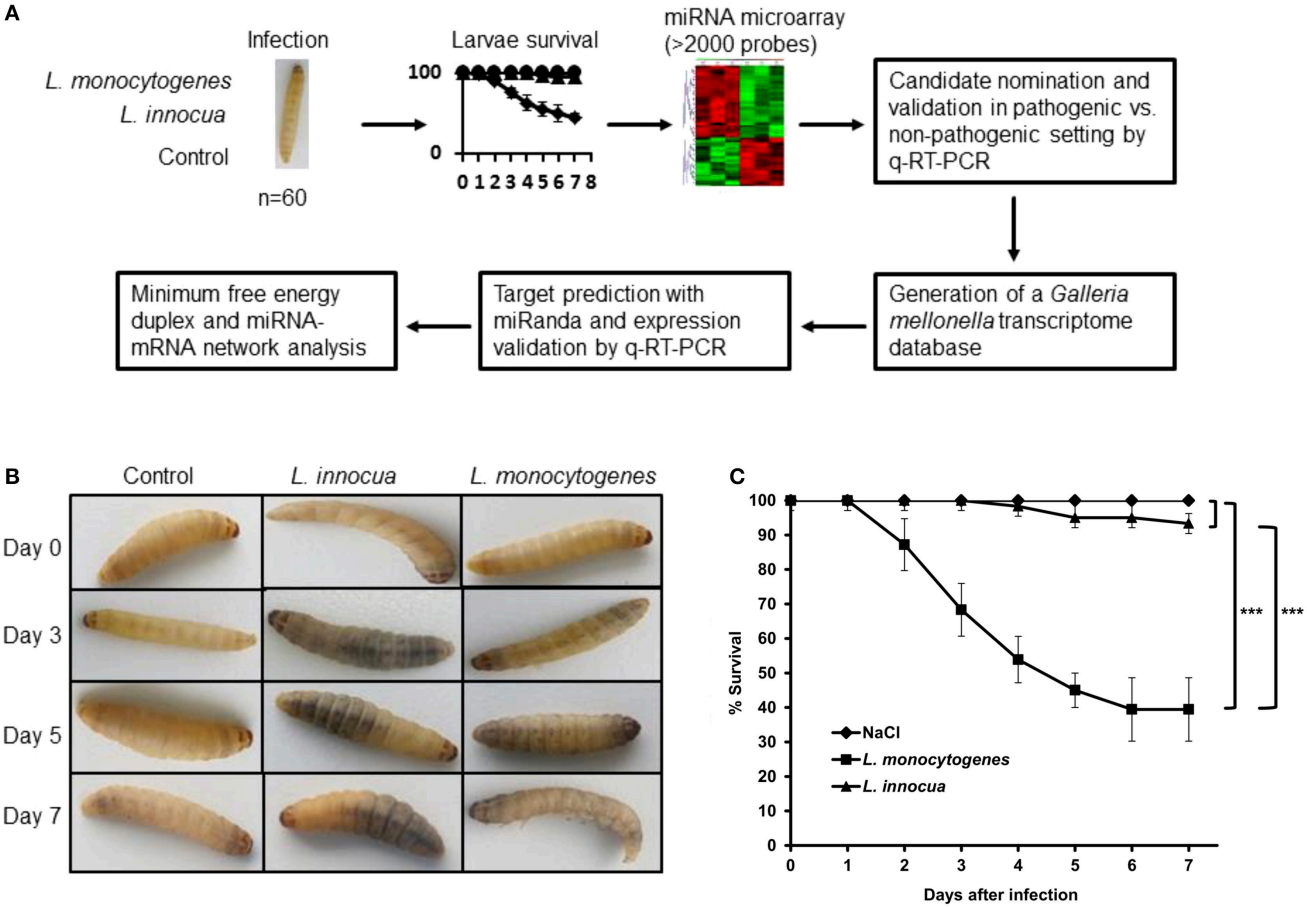
**(A)** Depicts the workflow taken in this study to comprehensively examine the miRNA response following infection of *G. mellonella* with pathogenic and non-pathogenic Gram-positive bacteria. **(B)** Macroscopic changes of *G. mellonella* larvae of infected larvae over time highlight the reduced viability of larvae infected with pathogenic *L. monocytogens*. **(C)** Survival curves of larvae (*n* = 60) infected with *L monocytogens, L. innocua* or control confirms macroscopic observations. This resulted in a median survival of 6 days post infection in in the *L. monocytogens* group (*p* ≤ 0.0001), while *L. inncoua* did not induce significant mortality (*p* > 0.05).

We used *G. mellonella* to systematically study the *in vivo* effect of infection with *L. monocytgenes* on the miRNA expression profile and downstream effect on their corresponding targets (Figure 1A). Infection with *L. monocytogenes* resulted in illness and decreased motility of larvae (Figure 1B), whereas non-pathogenic *L. innocua* had no effect on these parameters. The median survival of larvae infected with *L. monocytogenes* was ~50% after 7 days, while those infected with *L. innocua* survived at least 7 days (*p* < 0.001; Figure 1C). In addition, we have determined the bacterial load of larva at day one and five post infection and observed that dying larva revealed a higher bacterial load compared to surviving larva as previously reported (Mukherjee et al., 2010; see Figure S3).

Together, these results indicated that infection of *G. mellonella* with either pathogenic (*L. monocytogenes*) or non-pathogenic (*L. innocua*) bacteria adequately reflected pathogenicity as observed in vertebrate *in vivo* models.

**Figure 2:**
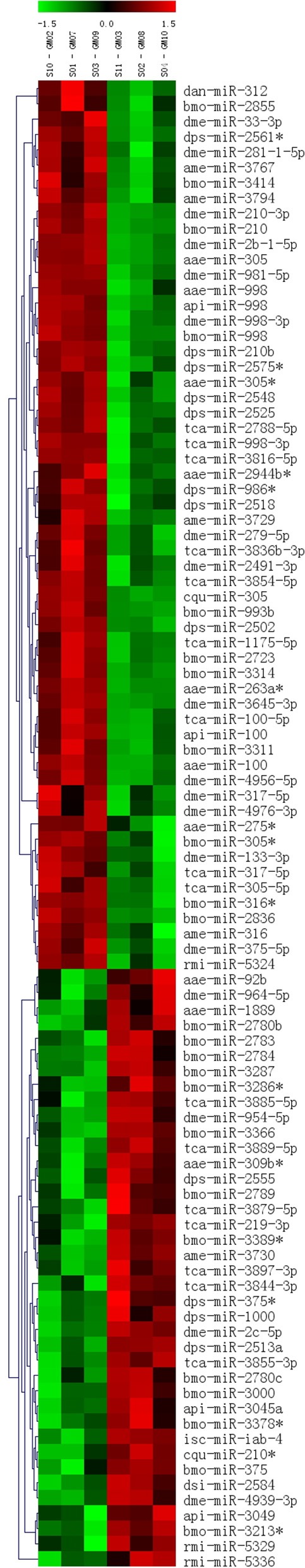
Heat map of miRNA microarray was generated between control and infected *G. mellonella*. This figure shows a set of statistically significant deregulated miRNAs upon infection with *L. monocytogenes* (*p* ≤ 0.01). Red, increased expression; Green, reduced expression. The lines in heat map: GM02, GM07, and GM09 represent control samples; GM03, GM08, and GM10 represent infected samples.

To examine the *in vivo* effect on the transcriptional profile of miRNAs induced by *L. monocytogens*, we isolated total RNA from *G. mellonella* larvae on the 5th day post infection and used a miRNA microarray. The microarray chip contains 2064 unique probes from different model insects such as *Bombyx mori* (559), *Drosophila melanogaster* (1539), *Tribolium castaneum* (394), *Apis melifera* (168) and *Acrythosiphon pisum* (103). The RNA samples of three independent experiments were tested. Compared to controls (saline injection) infection with *L. monocytogenes* resulted in alterations of signal intensities of 919 miRNAs, of which 90 (39 upregulated, 51 downregulated) were significantly deregulated (*p* < 0.01; Figure 2; Data Sheets S1, S2). It is notable, that a subset of miRNAs represented on the microarray is conserved between insect species and was therefore measured multiple times.

### *In vivo* deregulation of miRNAs occurs in a virulence-dependent fashion

**Figure 3:**
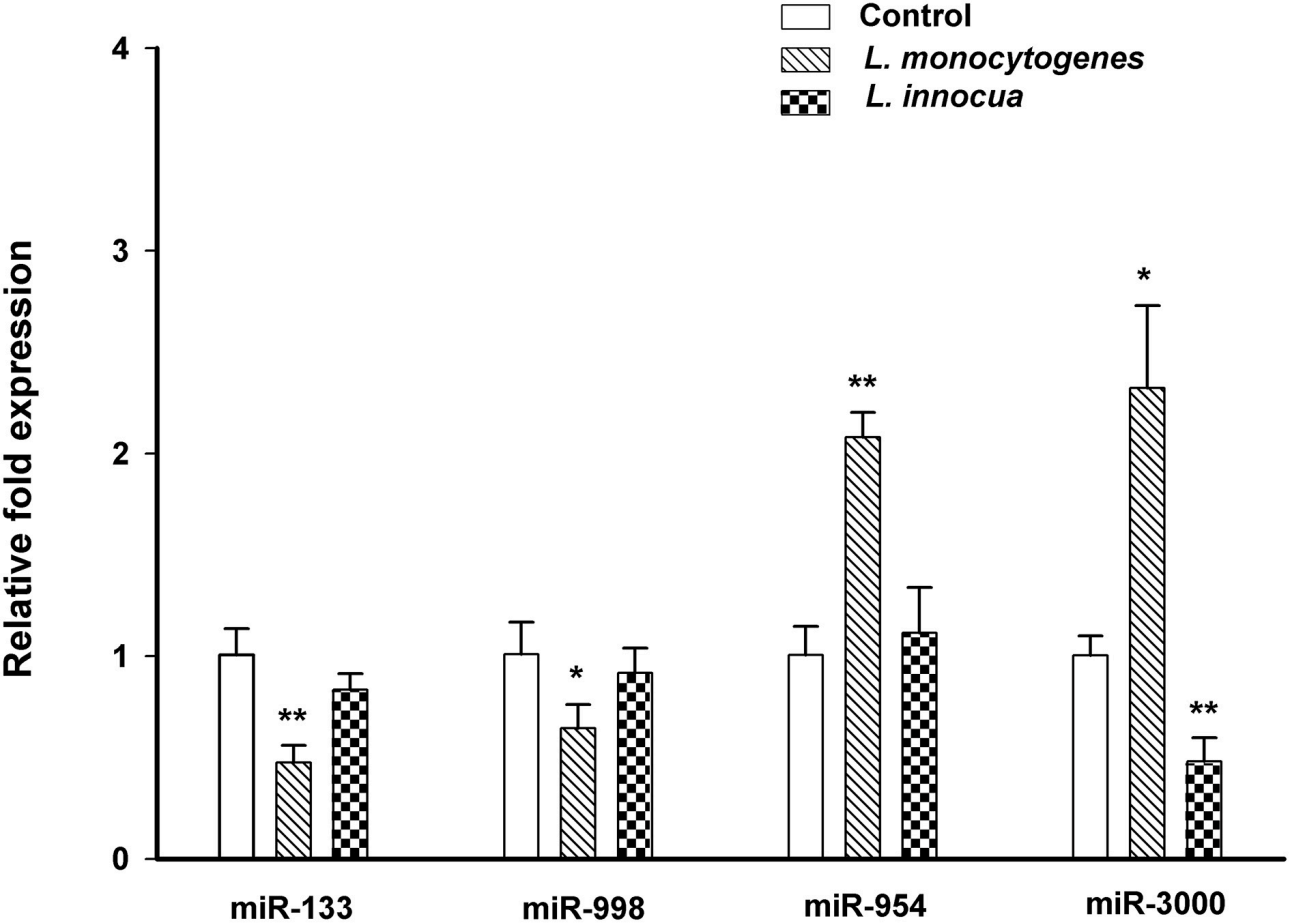
Validation of miRNA microarray analysis and patho/non-pathogenic mediated miRNA response in *G. mellonella*. In support of microarray, qRT-PCR analysis of miRNA with infection of *L. monocytogenes* showed significant reduced expression of miR-133 and miR-998 and increased expression of miR-954 and miR-3000. Upon infection with non-pathogenic *L. innocua* there is no significant changes in alteration of miRNA expression, except bmo-miR-3000, which is significantly downregulated. Error bars indicate standard deviations (^*^p ≤ 0.05; ^**^p ≤ 0.01).

We next used qRT-PCR to validate a subset of miRNAs that were significantly deregulated, have homologs in human and/or mouse (Ibáñez-Ventoso et al., 2008) and a known function *in vivo* (Ma et al., 2011; Saito et al., 2011). MicroRNA candidates were selected according to the following criteria: significantly deregulated expression pattern including consistency among replicates, signal intensity above 500 and a high degree of conservation among vertebrates and invertebrates. A total of 10 miRNAs (5 miRNAs from upregulated group and 5 miRNAs from downregulated group that were deregulated when compared to control samples) were tested for the validation of the microarray results. Among these miRNAs, dme-miR-133-3p, dme-miR-998-3p, dme-miR-954-5p, and bmo-miR-3000 were in excellent agreement with the microarray results (*R*^2^ > 0.99) (Figure S1). miRNAs dme-miR-133-3p, dme-miR-998-3p were significantly downregulated, whereas dme-miR-954-5p and bmo-miR-3000 were significantly upregulated upon infection with *L. monocytogenes*. We recently showed that in an *in vitro* model, miRNA deregulation depends on virulence-defining factors of *L. monocytogenes* (Izar et al., 2012). Another report showed that in *G. mellonella* that expression of miR-263a was reciprocally regulated comparing entomopathogen *Serratia entomophila* and non-pathogenic *E. coli* (Mukherjee and Vilcinskas, 2014). We therefore wished to investigate differential regulation of miRNAs after infection with pathogenic *L. monocytogens* and non-pathogenic *L. innocua*. Compared to *L. monocytogens, L. innocua* lacks several key virulence factors, including *Listeria* pathogenicity island 1 (LIPI-1), InlA, InlB and other surface–exposed proteins (Glaser et al., 2001). Indeed, we found that *L. innocua* failed to deregulate dme-miR-133-3p, dme-miR-998-3p, dme-miR-954-5p, and induced reciprocal regulation of bmo-miR-3000, which was downregulated when compared to *L. monocytogens* infection (Figure 3).

### Generation of a publically available annotated *G. mellonella* transcriptome database for miRNA target mRNA prediction

We next sought to predict putative targets of the significantly deregulated and validated miRNAs. We therefore generated an *in silico G. mellonella* transcriptome by collecting the ESTs of *G. mellonella* RNA-seq (454 and Illumina sequencing) from NCBI along with 18,690 pre-assembled contigs from Vogel et al. (2011). After quality trimming and normalization, a total of 25,196,088 RNA-seq reads, 12,057 ESTs, and 18,690 contigs were used for the assembly (see section Materials and Methods). The Trinity assembler produced 60,288 sequences. The number of assembled sequences of the Velvet/Oases assembly ranged from 125,562 (k-mer = 19) to 33,860 (k-mer = 75). In total 1,909,841 sequences were screened for coding regions. About 36 % (692,004 transcripts) of the sequences contained potential CDS. Clustering of the protein sequences produced 34,404 clusters. With the automatic functional annotation of the filtered cluster sequences, using different databases 60% (20,926) of the sequences could be annotated. For this purpose, we performed blastp searches in KEGG (Ogata et al., 1999), COG (Tatusov et al., 2003), Swissprot (Boutet et al., 2007), InterProScan (Jones et al., 2014), HMMER (Eddy, 2009), and searched against Pfam (Finn et al., 2014). The transcriptome database is available as a public SAMS project under the following URL:

https://www.uni-giessen.de/fbz/fb08/Inst/bioinformatik/Research/Supplements/galleria.

### *In silico* prediction of miRNA targets and stability of miRNA/mRNA duplexes indicate a virulence-dependent regulation of gene transcripts of the innate immunity

**Figure 4:**
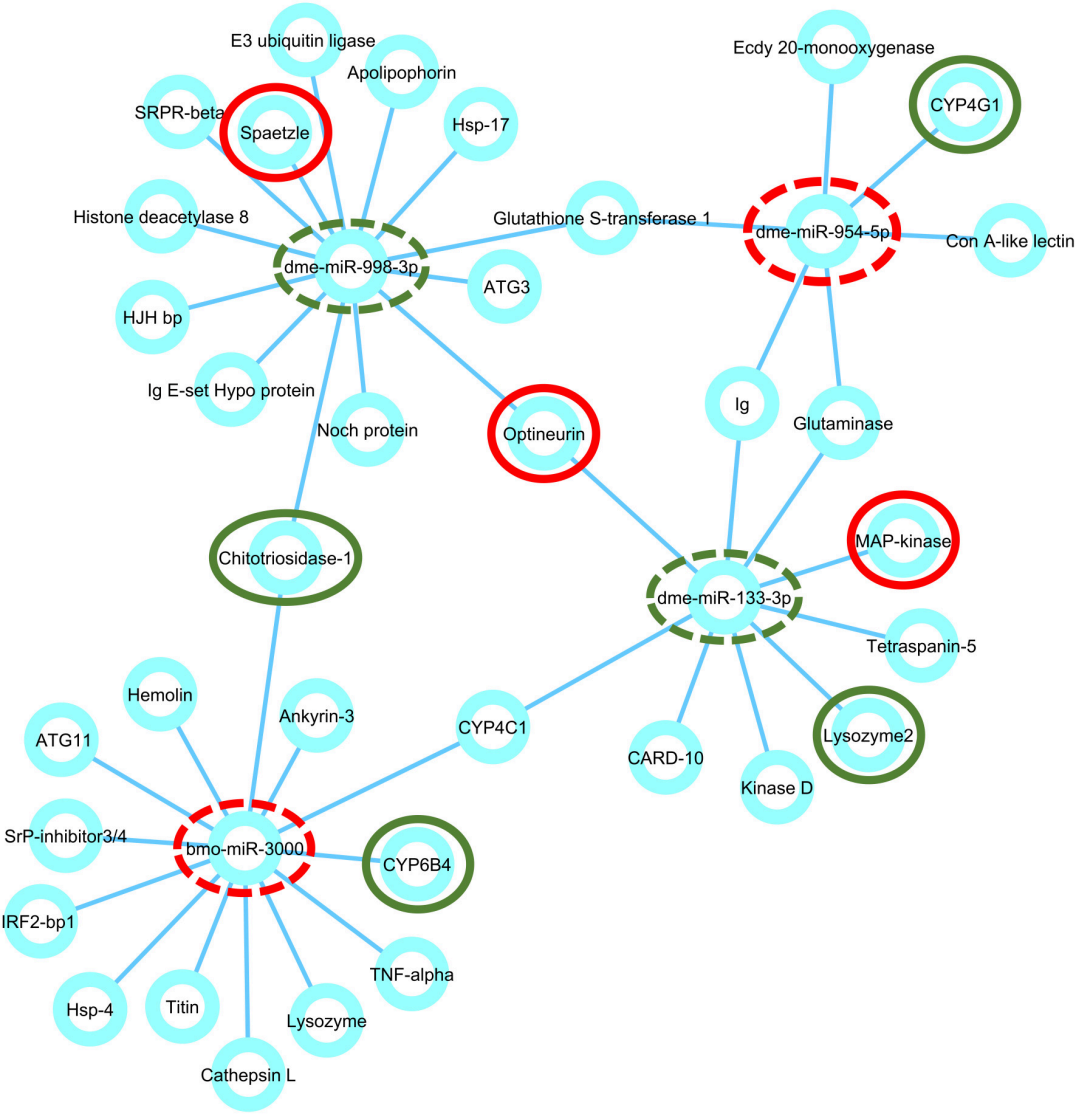
miRNA-mRNA network shown by cytoscape: After generating a *G. mellonella* reference transcriptome, we used miRanda to predict targets for validated miRNAs (miR-998, miR-133, miR-954, and bmo-3000) which were implemented in this network. The figure shows a network of selected targets for each miRNA. miRNAs are highlighted with dashed circles and target genes, which were subsequently tested for minimum free energy duplexes and validated by qRT-PCR, are highlighted with fully closed circles. Red color indicates increased expression and green color indicates reduced expression of the particular miRNA or mRNA in response to *L. monocytogenes* infection.

**Figure 5:**
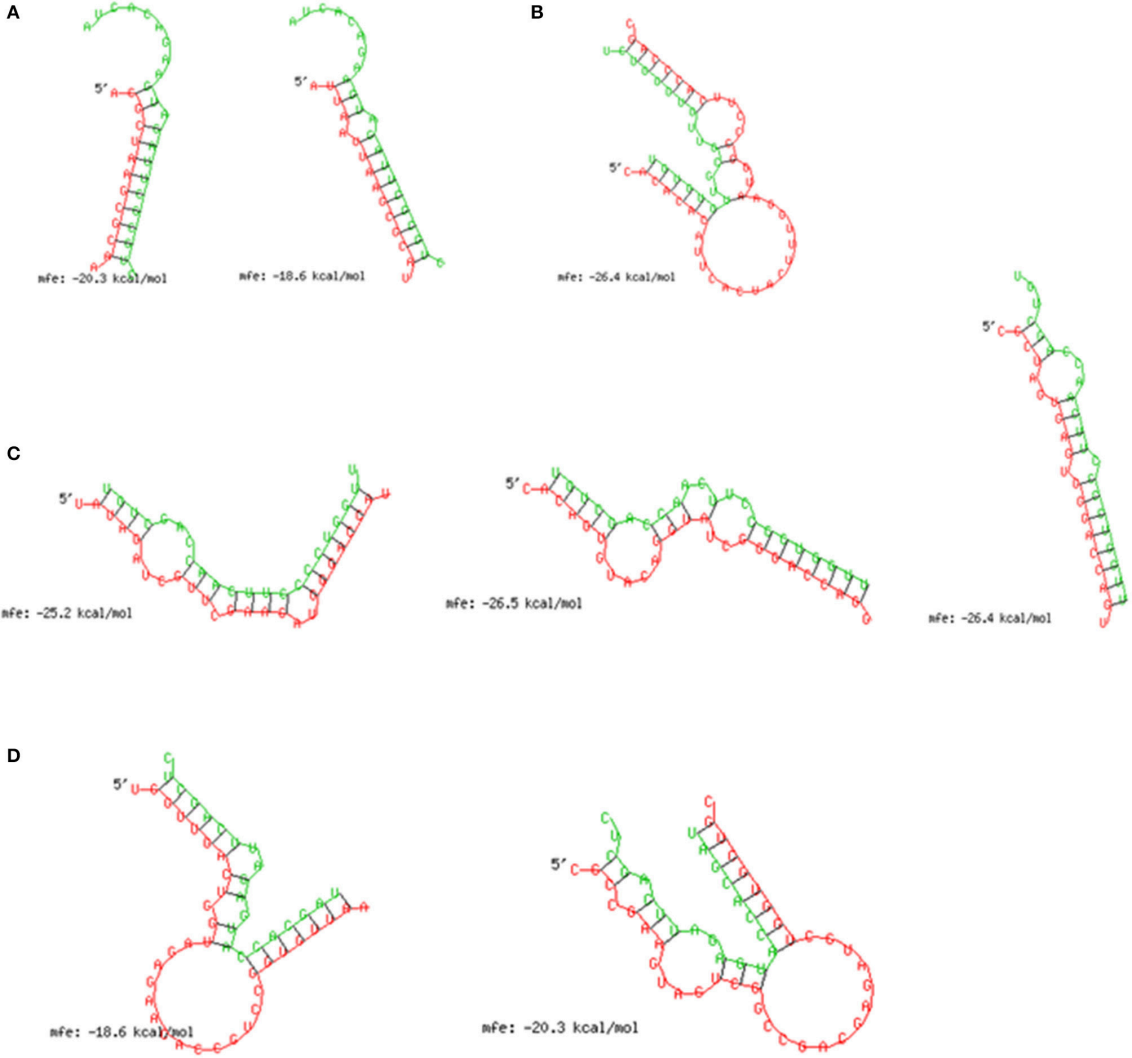
Depicts the minimum free energy duplexes of four selected microRNAs. Each figure shows the duplex of miRNAs and the 3′-UTR of target mRNAs (marked as 5′) of *G. mellonella*. The alignment shows the total miRNA sequence and the seed region it hybridizes to in the target 3′-UTR. **(A)** Duplex of bmo-miRNA-3000 and chitotriosidase-1 (left) and CYP6B4 (right); **(B)** dme-miR-954-5p CYP4G1; **(C)** dme-miR-133-3p and optineurin (left), MAP kinase (middle), and lysozyme2 (right); and **(D)** dme-miR-998-3p and optineurin (left) and spätzle (right).

A histogram of the predicted 3′ UTR lengths is shown in Figure S2. About 64.7% (22,265) of the sequences could be assembled with potential 3′ UTR. *In silico* miRNA target prediction for 4 miRNAs (dme-miR-954-5p, bmo-miR-3000, dme-miR-998-3p, and dme-miR-133-3p) with those 3′ UTR sequences provided 1,822 potential targets. The total list of target genes along with their corresponding gene ontology is summarized in Table S3. Furthermore we looked for an enrichment of gene ontologies associated to immunity using GOstats (Falcon and Gentleman, 2007). This analysis revealed that immunity related genes (GO:0002700, GO:0002922, GO:0002699, GO:0050776, GO:0002376, GO:0006959, GO:0002385, *p* ≤ 0.022) are significantly overrepresented in *Galleria mellonella* after infection with *L. monocytogenes* (see Table S4). This warrants further investigation, because the 255 overrepresented genes related to these GO number just correspond to a small portion (13%) of all genes associated with these immunity GO categories. From the list of targets we selected those that have known or predicted functions in host defense system against bacterial infections and visualized them in a miRNA-mRNA regulatory network using cytoscape (Figure 4). For example, bmo-miR-3000 was predicted to target chitotriosidase-1 and cytochrome P450 6B4 (CYP6B4) and cytochrome P450 4G1 (CYP4G1) was identified as a putative target of dme-miR-954-5p. Another remarkable putative target, optineurin was predicted hybridizing with both dme-miR-133-3p and dme-miR-998-3p. dme-miR-133-3p putatively targets MAP kinase transcripts and spätzle was found to be a target of dme-miR-998-3p. To assess the stability of predicted miRNA-mRNA interactions, we estimated minimum free energy level of miRNA-mRNA duplexes using RNAhybrid (Rehmsmeier et al., 2004). This tool provides energetically favorable sites for miRNA and its target transcript and takes into account potential intra-molecular hybridization within the target mRNA (Rehmsmeier et al., 2004). The optimal duplexes of selected miRNAs and predicted targets are shown in Figure 5.

### Concordant expression of miRNAs and putative target transcripts

**Figure 6:**
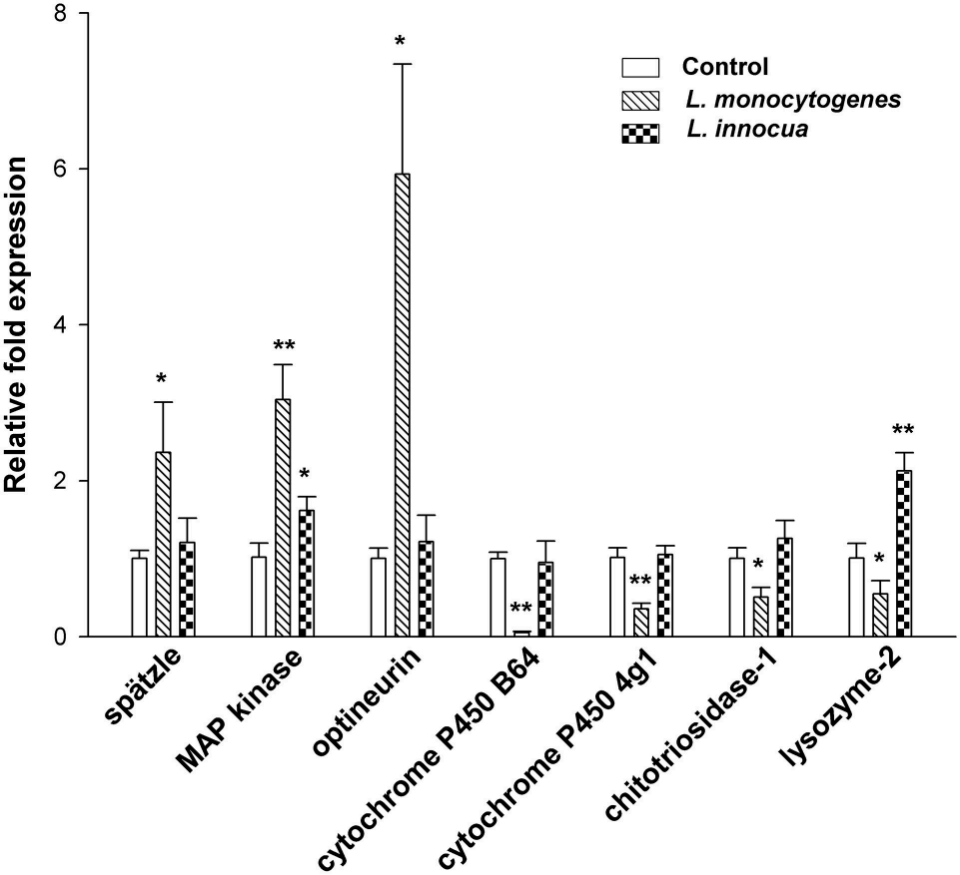
qRT-PCR analysis of predicted target genes expression upon infection with *L. monocytogenes* and *L. innocua*. With infection of *L. monocytogenes* target genes chitotriosidase-1 and lysozyme2 are showing significant reduced expression and also CYP6B4 and CYP4G1 are affected in expression. The factors involved in immune signaling pathways such as spätzle and MAP kinase, and autophagy receptor optineurin are significantly upregulated in *G. mellonella*. All these target genes are not affected with infection of *L. innocua*, except lysozyme2 and MAP kinase which are significantly upregulated. Error bars indicate standard deviations (^*^p ≤ 0.05; ^**^p ≤ 0.01).

To validate the results of the *in silico* miRNA target predictions, we performed real-time PCR to determine the expression levels of target genes and to correlate the miRNA with corresponding mRNA responses after infection with *L. monocytogenes* and *L. innocua*, respectively (Figure 6). Overall, downregulation of miRNAs is in line with increased levels of the corresponding target transcripts (Figure 6). For example, we found upregulation of optineurin, spätzle and MAP-kinase, while their regulating miRNAs dme-miR-133-3p and dme-miR-998-3p were downregulated. We found that increased levels of bmo-miR-3000 and dme-miR-954-3p were associated with decreased mRNA levels of chitotriosidase-1, CYP6B4 and CYP4G1, respectively. A subset of mRNAs was predicted to be targeted by two of the examined miRNAs, such as optineurin, which is regulated by dme-miR-133-3p and dme-miR-998-3p, which might result in the strongly elevated mRNA levels of this gene product. Chitotriosidase-1 is targeted by inversely transcribed dme-miR-998-3p and bmo-miR-3000. Interestingly, the level of the chitotriosidase-1 mRNA appeared to be regulated in an integrated fashion, indicating that multiple miRNAs may be involved in the fine tuning of the same target transcript. We also found discordant regulation of one miRNA (dme-miR-133-3p) and its target transcript (lysozyme2). In addition to other post-transcriptional regulatory mechanisms, it is possible that an unmeasured miRNA may regulate this target transcript in an integrated fashion as observed for chitotriosidase-1. We next examined the transcriptional output of these target genes following infection with *L. innocua*. Consistent with the absence of significant deregulation of corresponding miRNAs, we did not find alterations in expression levels of spätzle or optineurin. Furthermore, lysozyme2 was upregulated despite the absence of direct regulation by miRNAs, further supporting the above mentioned observation that this gene product may be regulated by multiple miRNAs that were unmeasured in this experiment. Overall, we find good concordance between predicted miRNA/mRNA interactions and supporting evidence for a virulence-dependent miRNA-mediated mRNA regulation in bacterial infections.

## Discussion

In this study, we used an insect-wide microarray containing 2064 probes to systematically examine the *in vivo* miRNAs expression profiles in the greater wax moth *G. mellonella* following infection with Gram-positive bacteria. We measured and validated significant deregulation of several miRNAs that occurred upon infection with pathogenic *L. monocytogenes*, but not with non-pathogenic *L. innocua*. To predict putative targets of these miRNAs, we compiled a *G. mellonella* transcriptome. We estimated the energetic miRNA/mRNA duplexes and validated target transcripts derived from the prediction analysis. Consistent with previous studies, our results indicate a specific virulence-dependent induction of miRNAs that occurred upon infection with *L. monocytogenes* but not in response to non-pathogenic *L. innocua*.

Infection of *G. mellonella* with *L. monocytogenes* induced upregulation of 39 and downregulation of 51 miRNAs. These findings have been validated for four selected miRNA (dme-miR-133-3p, dme-miR-998-3p, dme-miR-954-5p, and bmo-miR-3000) using quantitative real-time PCR. A sequence homology study of known miRNA between *C. elegans, D. melanogaster* and human showed significant conservation of miRNAs indicating that miRNAs dme-miR-998-3p and dme-miR-133-3p are conserved as miR-29 and miR-133 in higher animals, respectively (Ibáñez-Ventoso et al., 2008). Both miRNAs and their respective homologs have been implicated in the response to infection and inflammation. Ma et al. showed that infection of NK cells and T cells with *L. monocytogenes* and *Mycobacterium bovis* led to downregulation of miR-29 which targets IFN-γ (Ma et al., 2011). Chronic infection with *Helicobacter pylori* led to downregulation of tissue specific miR-133 miRNA, increased expression of acute phase proteins (Saito et al., 2011). In line with these studies, we observed significant downregulation of miR-998 and miR-133 and increased levels of their targets spätzle and optineurin, indicating a conserved role of this miRNA and its homologs in the response to bacterial infections.

In previous work, we investigated the role of different miRNAs during infection of *L. monocytogenes* in the epithelial Caco-2 cell line and showed that expression of miR-16 and miR-146b depends on major virulence factors such as thiol activated toxin hemolysin (listerolysin) and internalins, a family of proteins that determine the ability to adhere and invade specific target cells (Izar et al., 2012). Subsequently, induction of miRNA deregulation by several pathogenic bacteria via virulence-factor dependent mechanisms has been shown in studies investigating infections with *Staphylococcus epidermidis, Salmonella enterica* serovar Typhimurium and *Yersinia pseudotuberculosis* (Siddle et al., 2015). Concordantly, our current study further supports the concept that miRNA deregulation is specific to the virulence of *L. monocytogens* rather than a non-specific response to bacteria, including non-pathogenic *L. innocua*. In detail, miRNAs dme-miR-954 and bmo-miR-3000 were upregulated whereas miR-133 and miR-998 were downregulated following a pathogenic *L. monocytogenes* infection. In response to *L. innocua* infection, no significant change of these miRNA expression levels was measured, except for bmo-miR-3000 which indeed exhibited an inverse expression profile. Together, these findings and previous observations strongly support a concept of virulence-dependent miRNA regulation during host-microbe interactions.

We investigated the potential biological implications of differentially deregulated miRNAs. To predict the target genes of aforementioned miRNAs, we have established a publically available database from all ESTs published in NCBI that were expressed under different stress responses in *G. mellonella*. Using miRanda (Enright et al., 2003), we predicted putative targets for above mentioned miRNAs, validated these by qRT-PCR and calculated the minimum free energy levels between mRNA-miRNA duplexes using the RNAhybrid tool (Rehmsmeier et al., 2004).

Host invasion by pathogens leads to activation of a number of signaling pathways of the innate immune response. In insects, for example, Gram-positive peptidoglycans and fungal glucans are recognized by a receptor of the toll pathway known as spätzle (Dionne and Schneider, 2008). Activation of the toll pathway results in the synthesis of antimicrobial peptides to counteract pathogens (Dionne and Schneider, 2008). In addition, several bacterial effector proteins are able to trigger the MAP kinase signaling pathway, which is pivotal in the innate and adaptive immunity of higher animals. *L. monocytogenes* activates MAP kinase by attaching to the cell surface of epithelial cells (Tang et al., 1994). In insects, MAP kinases are involved in the activation of prophenoloxidase, in turn which induce phagocytosis and melanization of hemocytes (Mavrouli et al., 2005). Here, we detected upregulation of spätzle and MAP kinase, putative targets of downregulated miR-998 and miR-133, respectively, after *L. monocytogenes* infection. The concordance of miRNA/mRNA regulation might indicates a role of this circuit in the activation of signaling pathways, synthesis of AMPs and the defense response of larvae against listerial infection. Optineurin is a receptor for autophagy and plays a major role in removal of intracellular bacteria (Wild et al., 2011; Puri et al., 2017). In agreement with previously described roles, we observed strong induction of optineurin, which is correlated with decreased expression of two regulatory miRNAs (miR-133 and miR-998). Together, this interaction is predicted to facilitate increased clearance of intracellularly localized pathogens by autophagy. The induction of these pathways again appears to be virulence-dependent, since we observed no deregulation of these miRNAs and the transcripts of their targets upon infection with *L. innocua*, with the exception MAP kinase which was upregulated to lesser extent. In contrast, clearance of *L. innocua* might be correlated with increased expression of lysozyme2, which was exclusively upregulated in this setting and downregulated in *L. monocytogens* infection. Similarly, chitotriosidase-1, the best characterized chitinase in mammals, is induced by pro-inflammatory cytokines, such as TNF-α and GM-CSF in response to bacterial and fungal pathogens (Lee et al., 2011). It is possible that repression of lysozyme2 and chitotriosidase-1 represents mechanisms of evading the host-response by pathogenic bacteria, while non-pathogenic pathogens, such as *L. innoc*ua are efficiently cleared via lysozyme2 activity. Indeed, we observed evidence that bmo-miR-3000 may actively be involved in this process, as we observed upregulation during infection with *L. monocytogenes* and downregulation upon infection with *L. innocu*a.

Infection with *L. monocytogenes* induced increased expression of miR-954 and miR-3000 and corresponding reduced expression levels of CYP6B4 and CYP4G1, respectively, while non-pathogenic *L. innocua* had no effects on these miRNA/mRNA pairs. Xenobiotic enzymes play major role in toxin and drug metabolism in multicellular organisms. Cytochrome P450 enzyme showed reduced expression upon infection with *L. monocytogenes* in mice hepatic tissue and brain, which may cause severe complication with drug metabolism (Armstrong and Renton, 1993; Garcia Del Busto Cano and Renton, 2003). Previous reports uncovered interactions between the xenobiotic metabolism and infection and inflammation processes induced by bacterial pathogens and other immunostimulants (Reynaud et al., 2008). In juvenile carp, for example, *L. monocytogenes* reduces activities of cytochrome P450 enzymes and ethoxyresorufin O-deethylase (Chambras et al., 1999). While the precise effect of cytochrome P450 enzymes on this infection model requires further investigation, it seems plausible that downregulation of the enzymes may improve pathogen survival. This regulation may be an actively induced process by *L. monocytogenes*, but not *L. innocua*, via miRNA-mediated degradation of the corresponding target transcripts. Finally, all these above mentioned interactions require further investigations to describe the precise mechanistic role of differentially regulated miRNAs and their putative targets in response to infection of *G. mellonella* with *L. monocytogenes*.

## Conclusion

**Figure 7:**
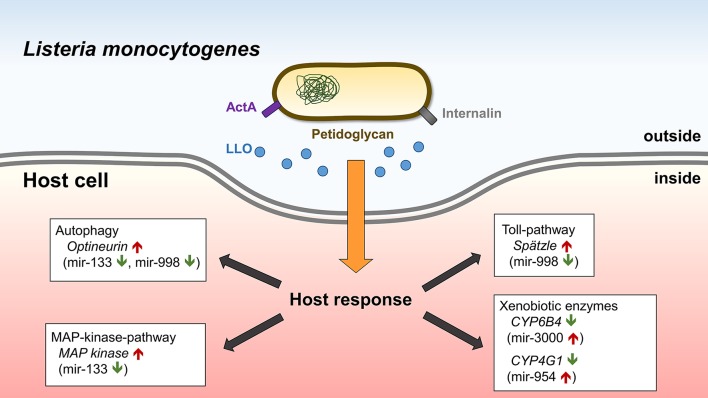
Graphical illustration of the microRNA response of *Galleria mellonella* to *L. monocyotogenes* infection.

In conclusion, this study demonstrates the feasibility of leveraging *G. mellonella* as an *in vivo* model to examine miRNA expression following infection with pathogenic and non-pathogenic bacteria. We used orthogonal approaches to determine miRNA expression, *in silico* algorithms to predict the occurence and energetic stability of miRNA-mRNA and direct validation of predicted targets. We uncovered miRNA/mRNA expression patterns specific to pathogenic *L. monocytogenes* compared to non-pathogenic *L. innocua*, which revealed the role of miRNAs in regulation of immune response (Figure 7). Homologues of miRNAs described in this study were shown to have important roles in mammalian infection models. Thus, *G. mellonella* represents a simple and valuable *in vivo* model capable of recapitulating the regulation of miRNAs in host-microbe interactions in higher animals.

## Author contributions

GM, TC, and TH conceived the work and designed the experiments; GM and OR performed the experiments; GM, BI, OR, AG, TC, and TH analyzed data; GM, BI, OR, TS, AG, TC, and TH wrote the paper.

### Conflict of interest statement

The authors declare that the research was conducted in the absence of any commercial or financial relationships that could be construed as a potential conflict of interest.

## Supplementary material

Figure S1 Correlation between the fold changes measured by qRT-PCR and microarray methods.

Figure S2 Length distribution of the predicted 3′-UTRs.

Figure S3 Bacterial load per larva after day 1 and 5 post infection with *L. innocua* and *L. monocytogenes*

Table S1 Total microarray analysis of miRNAs with infection of *L. monocytogenes*.

Table S2 List of primers used in this study.

Table S3 miRanda target prediction and transcript annotation.

Table S4 Gene ontology analysis using GOstats to determine overrepresented gene ontology categories related to immune response.

Data Sheet S1 Microarray signal intensities analysis.

Data Sheet S2 Microarray statistical analysis.
