# Supplementary material for: Listeria monocytogenes Induces a Virulence-Dependent microRNA Signature That Regulates the Immune Response in Galleria mellonella
Source: Front Microbiol. 2017 Dec 12;8:2463. doi: 10.3389/fmicb.2017.02463 (PMC5733040; doi:10.3389/fmicb.2017.02463)
Supplement: Figure S1 — Correlation between the fold changes measured by qRT-PCR and microarray methods. [file Image1.PDF]

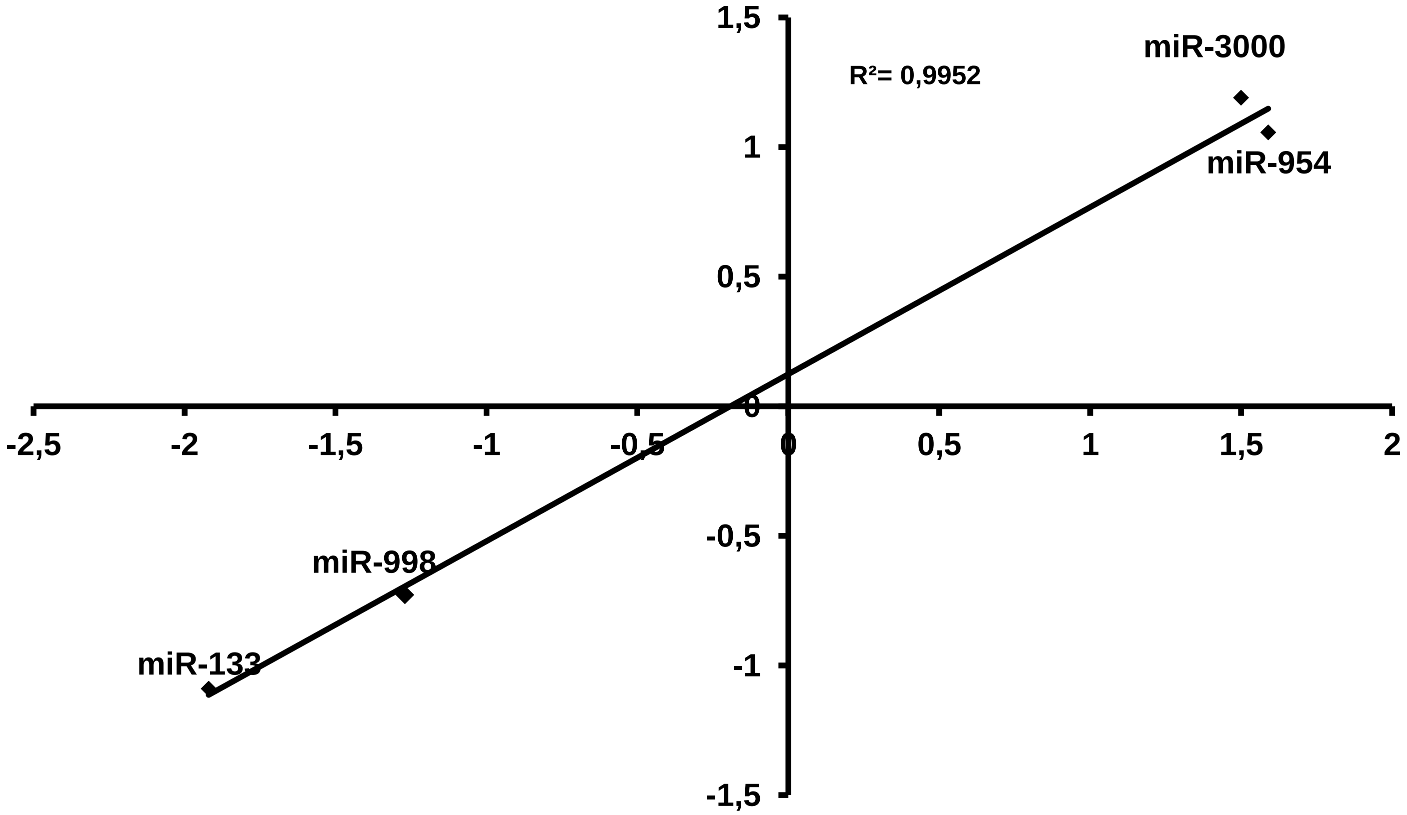

Supplementary Fig 1: Correlation between the fold changes measured by qRT-PCR and microarray methods. The figure shows log2fold changes of miR-133, miR-998, miR-954 and miR-3000 in infected *G. mellonella*. The strong correlation R^2= 0,9952 is achieved between the fold changes measured by both independent techniques.

Mannala *et al.,* Fig.S1
