# Supplementary material for: Listeria monocytogenes Induces a Virulence-Dependent microRNA Signature That Regulates the Immune Response in Galleria mellonella
Source: Front Microbiol. 2017 Dec 12;8:2463. doi: 10.3389/fmicb.2017.02463 (PMC5733040; doi:10.3389/fmicb.2017.02463)
Supplement: Figure S2 — Length distribution of the predicted 3′-UTRs. [file Image2.PDF]

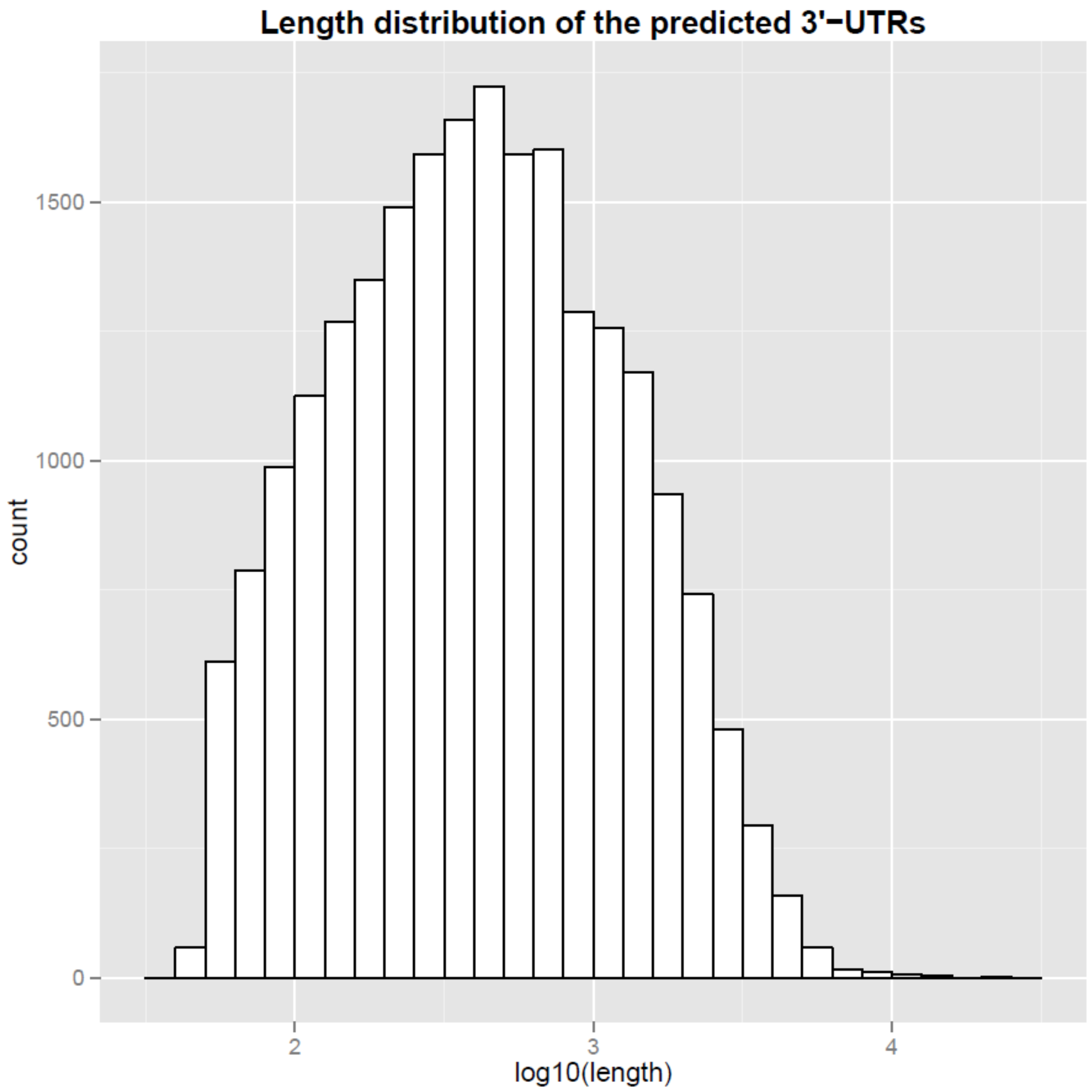

Supplementary Fig 2: Histogram that represents the distribution of the predicted 3'-UTR lengths on the assembled transcripts. Coding regions (CDS) on the transcripts were predicted using the Trans Decoder tool. The complete sequence after the predicted stop-codon to the end of the transcript was used as 3'-UTR for microRNA target prediction.
