# Supplementary figures and images for: Listeria monocytogenes Induces a Virulence-Dependent microRNA Signature That Regulates the Immune Response in Galleria mellonella
Source: Front Microbiol. 2017 Dec 12;8:2463. doi: 10.3389/fmicb.2017.02463 (PMC5733040; doi:10.3389/fmicb.2017.02463)

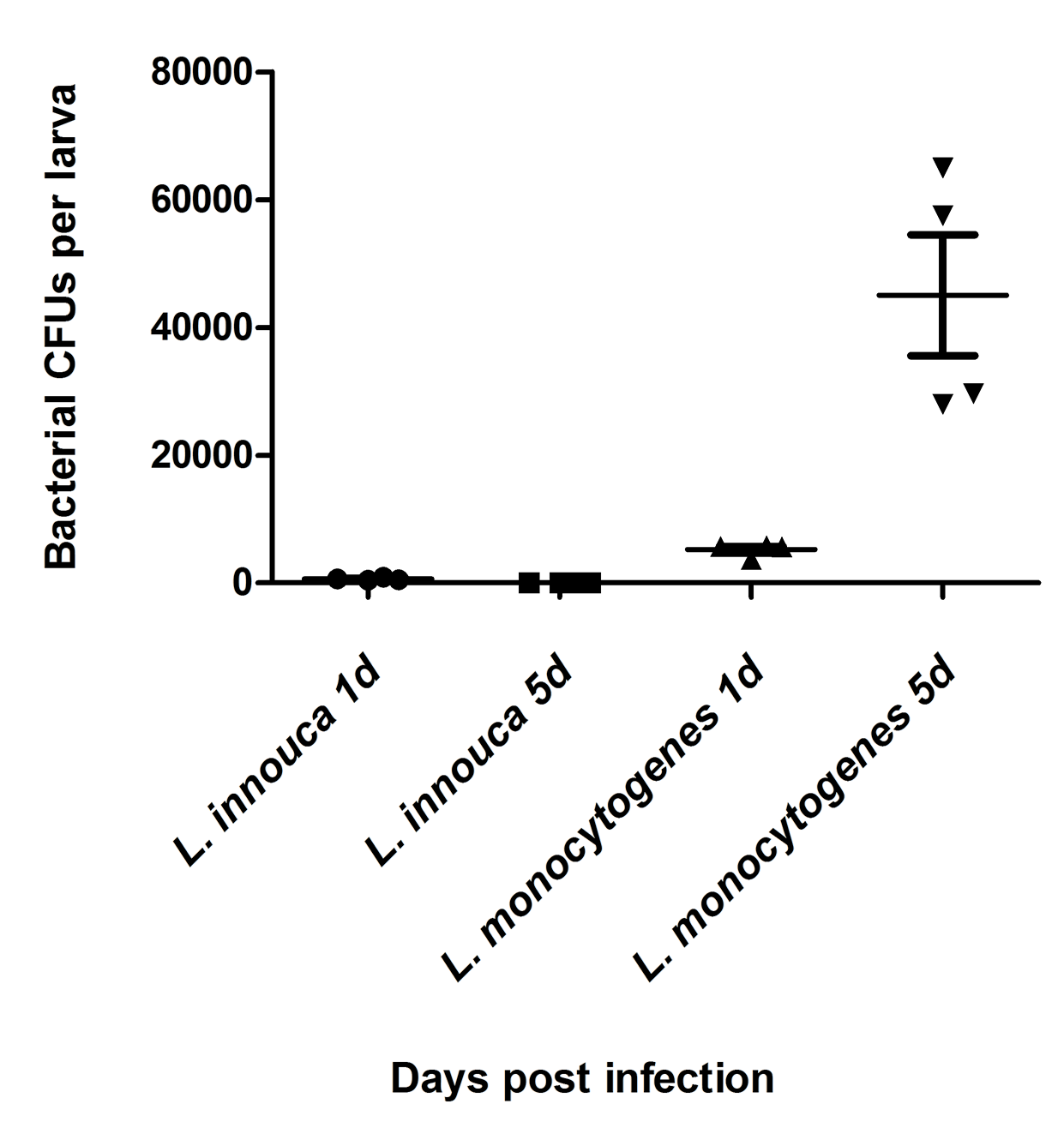

Supplement: Figure S3 — Bacterial load per larva after day 1 and 5 post infection with L. innocua and L. monocytogenes [file Image3.TIF]
