# Supplementary material for: Listeria monocytogenes Induces a Virulence-Dependent microRNA Signature That Regulates the Immune Response in Galleria mellonella
Source: Front Microbiol. 2017 Dec 12;8:2463. doi: 10.3389/fmicb.2017.02463 (PMC5733040; doi:10.3389/fmicb.2017.02463)
Supplement: Table S2 — List of primers used in this study. [file Table2.PDF]

List of primers used in this study

Primers for quantification of target mRNAs	
**Target gene**	**Sequence (5´-3´)**
chitotriosidase-1 Fwd	TGTTGCAGCTCCAGAAACAC
chitotriosidase-1 Rev	TGATGTTGAGGGTTGCCATA
cytochrome P450 6B4 Fwd	GGATAAAGCGGTCAGTTTGC
cytochrome P450 6B4 Rev	TCGAATAAGGCACCCAACAT
cytochrome P450 4g1 Fwd	TTGGGATCATTGGGTTGATT
cytochrome P450 4g1 Rev	GCCAGCTCTTTGTCAACCTC
spätzle Fwd	CACAGAACGAGTCGCTGCTA
spätzle Rev	TCGGGGTACACAACCTTCTC
optineurin Fwd	CGATATACGACCGCAGGTTT
optineurin Rev	GGCCTCTCCCCTTAATATGC
MAP kinase Fwd	GAGCAACAACCCCACAGATT
MAP kinase Rev	TCCTGAATTCCCTCTTGTGG
lysozyme 2 Fwd	TGTGTGGAGCCCAGAATACA
lysozyme 2 Rev	TGCCAGTCCTGGAATTAGGT
18S rRNA Fwd	TCTCAAAGATTAAGCCATGC
18S rRNA Rev	CTGAGTAAGATCTAAGGAACC

Primer sequences used for quantification of miRNAs	
**miRNA**	**Sequence (5´-3´)**
dme-miR-954-5p	UCUGGGUGUUGCGUUGUGUGU
bmo-miR-3000	CUGCGCUUAGAUGAAGACACUA
dme-miR-998-3p	UAGCACCAUGAGAUUCAGCUC
dme-miR-133-3p	UUGGUCCCCUUCAACCAGCUGU
dme-miR-307a-3p	UCACAACCUCCUUGAGUGAG
