# Supplementary material for: Listeria monocytogenes Induces a Virulence-Dependent microRNA Signature That Regulates the Immune Response in Galleria mellonella
Source: Front Microbiol. 2017 Dec 12;8:2463. doi: 10.3389/fmicb.2017.02463 (PMC5733040; doi:10.3389/fmicb.2017.02463)
Supplement: Data Sheet S1 — Microarray signal intensities analysis. [file DataSheet1.PDF]

Microarray signal intensities analysis

Standard data analysis includes the determination of detectable signals, calculation of signal intensities, and calculation of differential ratios. The data process begins with background subtraction, Cy3/Cy5 channel normalization, detectivity determination, and then p-value calculation.

Background Subtraction

Background is determined using a regression-based background mapping method. The regression is performed on 5% to 25% of the lowest intensity data points excluding blank spots. Raw data matrix is then subtracted by the background matrix.

Normalization

Normalization is carried out using a LOWESS (Locally-weighted Regression) method on the background- subtracted data.[1] The normalization is to remove system related variations, such as sample amount variations, different labeling dyes, and signal gain differences of scanners so that biological variations can be faithfully revealed.

Detectivity Determination

A transcript to be listed as detectable must meets at least two conditions: signal intensity higher than 3× (background standard deviation) and spot CV < 0.5. CV is calculated by (standard deviation)/ (signal intensity). When repeating probes are present on an array, a transcript is listed as detectable only if the signals from at least 50% of the repeating probes are above detection level.

p-Value Calculation

After the normalization, the p-values of the difference between Cy3 and Cy5 signals are calculated as following. Let $StdCy3$ and $StdCy5$ be the standard deviations Cy3 and Cy5 probe areas, respectively. Let $Cy3$ and $Cy5$ be the signal intensities (the values obtained after background subtraction and normalization) of the Cy3 and Cy5 probe areas. And let $StdBkgCy3$ and $StdBkgCy5$ be the standard deviations of background values of Cy3 and Cy5 channels. Let

$$C = \sqrt{StdBkgCy3^2 + StdBkgCy5^2}$$
$$dferr = \sqrt{StdCy3^2 + StdCy5^2} + C$$

where C is a numeral number and $dferr$ is a 1D array of size n. Let

$$Stemp1 = \frac{Cy5 - Cy3}{dferr}$$
$$Stemp2 = \frac{Stemp1 - mean(Stemp1)}{\sqrt{2}}$$

and

$$p(i) = \frac{2}{\sqrt{\pi}} \int_{Stemp2(i)}^{\infty} e^{-t^2} dt$$

If $p(i) < 0.01$, it is plotted as red spot in a log scatter plot.

1 B. M. Bolstad, R. A. Irizarry, M. Astrandand T. P. Speed, (2003) "A comparison of normalization methods for high density oligonucleotide array data based on variance and bias", Bioinformatics, 19 (2), 185-193.
